# Design Parameters to Control Synthetic Gene Expression in *Escherichia coli*

**DOI:** 10.1371/journal.pone.0007002

**Published:** 2009-09-14

**Authors:** Mark Welch, Sridhar Govindarajan, Jon E. Ness, Alan Villalobos, Austin Gurney, Jeremy Minshull, Claes Gustafsson

**Affiliations:** 1 DNA2.0, Menlo Park, California, United States of America; 2 OncoMed Pharmaceuticals, Inc., Redwood City, California, United States of America; University of Edinburgh, United Kingdom

## Abstract

**Background:**

Production of proteins as therapeutic agents, research reagents and molecular tools frequently depends on expression in heterologous hosts. Synthetic genes are increasingly used for protein production because sequence information is easier to obtain than the corresponding physical DNA. Protein-coding sequences are commonly re-designed to enhance expression, but there are no experimentally supported design principles.

**Principal Findings:**

To identify sequence features that affect protein expression we synthesized and expressed in *E. coli* two sets of 40 genes encoding two commercially valuable proteins, a DNA polymerase and a single chain antibody. Genes differing only in synonymous codon usage expressed protein at levels ranging from undetectable to 30% of cellular protein. Using partial least squares regression we tested the correlation of protein production levels with parameters that have been reported to affect expression. We found that the amount of protein produced in *E. coli* was strongly dependent on the codons used to encode a subset of amino acids. Favorable codons were predominantly those read by tRNAs that are most highly charged during amino acid starvation, not codons that are most abundant in highly expressed *E. coli* proteins. Finally we confirmed the validity of our models by designing, synthesizing and testing new genes using codon biases predicted to perform well.

**Conclusion:**

The systematic analysis of gene design parameters shown in this study has allowed us to identify codon usage within a gene as a critical determinant of achievable protein expression levels in *E. coli*. We propose a biochemical basis for this, as well as design algorithms to ensure high protein production from synthetic genes. Replication of this methodology should allow similar design algorithms to be empirically derived for any expression system.

## Introduction

Protein expression is important at many different levels of biological research. The cost of production for biopharmaceuticals and recombinant research reagents depends in large part upon the protein expression levels that can be achieved; construction of metabolic pathways requires that genes moved from one organism express protein in another; even biochemical studies of fundamental processes are frequently hampered or made impossible because sufficient amounts of protein cannot be obtained.

Genetic constructs for the expression of proteins now frequently use synthetic DNA. This is because sequence information from genome and metagenome sequencing projects has increased exponentially over the last decade [1], but most of these sequences are not available as physical DNA. The increase in speed and decrease in cost of synthetic DNA provides a convenient route to obtain genes encoding these virtual proteins [2]. Modifications of the natural DNA sequences are often introduced into the synthetic genes with the aim of enhancing expression, particularly in heterologous hosts.

Designing a gene to express a protein requires choosing from an enormous number of possible DNA sequences [3]. Most current synthetic gene design strategies are guided by mimicry of natural gene characteristics thought to be relevant for increased expression [4]. A variation on this approach is to copy the codon bias of a subset of highly-expressed native host genes [5] or even to exclusively use the codons most common in highly expressed genes [6]. The codon bias of a gene toward common codons is reflected in the Codon Adaptation Index (CAI) [7]. While genes designed to match host bias or maximizing CAI have expressed successfully in many instances [8], [9], clear relationships between these practices and expression are lacking. Most reported “codon optimization” successes describe only two genes: one natural and one synthetic [8]. Since only successful optimization experiments are published, and published examples generally differ in many respects, one cannot draw reliable conclusions on how best to design synthetic genes [3], [9], [10].

A recent study of expression of a diverse library of GFP genes in *E. coli* concluded that expression was limited primarily at initiation of translation [11]. Impaired expression correlated with a strong mRNA secondary structure near the translational start site, but no dependence on CAI or overall GC content was observed. However, a significant body of literature suggests that synonymous codon usage beyond the initiation region can impact expression [3], [8], [9]; for example *E. coli* strains over-expressing rare tRNAs can significantly improve gene expression [12]–[14].

In this work we have examined the relationship between protein expression and gene sequence characteristics, using two different proteins of commercial value for which expression levels were limiting. We designed and independently synthesized about 40 genes encoding each of these proteins; synonymous codon variation caused more than 40-fold variation in expression. We identified sequence properties that correlated with expression by combining partial least squares regression [15], [16] with genetic algorithms [17]. Variation among both gene sets was highly correlated to the codon biases for 10 amino acids. We tested the predictive value of these correlations by designing and testing several new genes; expression levels of these genes were high and well predicted. Finally, we discuss a possible biochemical basis for the codon preferences we observe.

## Results

### Synonymous substitutions cause expression differences

Two genes were chosen as targets for systematic exploration of the effect of synonymous codon usage on expression; one encoding the DNA polymerase of *Bacillus* phage Φ29 [18], the second encoding a synthetic single-chain antibody fragment (scFv) developed by OncoMed, Redwood City, CA. These genes were selected because they encode evolutionarily, structurally and functionally different proteins. There was also immediate commercial value in improving their expression as well as expression of the general classes they represent.

Two initial variant sets were designed: 21 variants of the polymerase gene and 24 of the scFv. Only synonymous codon usage within the open reading frame was varied. Gene variants were designed by back-translating the protein sequence using a Monte Carlo repeated random sampling algorithm to select codons probabilistically from codon frequency lookup tables [19]. Different sequences were obtained from different lookup tables, and by independently controlling predicted mRNA structures and GC bias in the first 15 codons (see Supplementary Material). The average pairwise DNA sequence identity was 79% and 82% within the scFv and polymerase variants, respectively.

**Figure 1:**
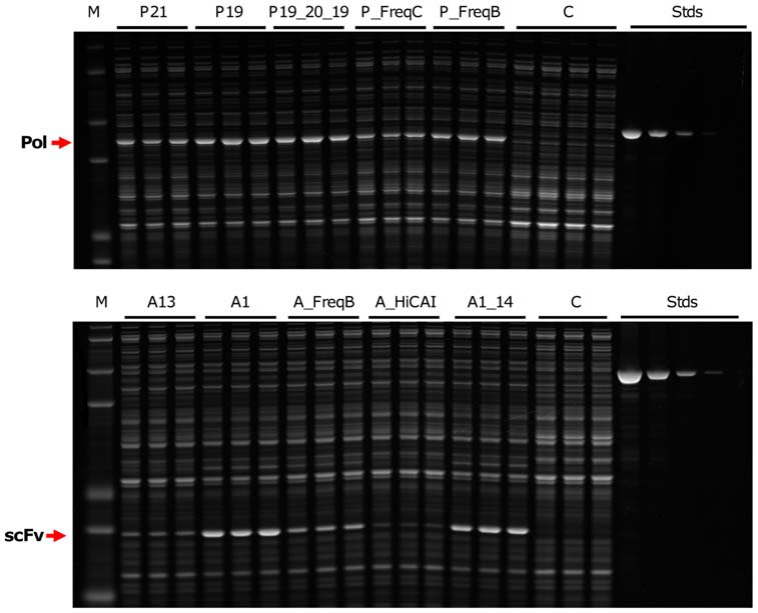
Protein expression from variant genes. Equal amounts of bacterial lysates were separated by polyacrylamide gel electrophoresis and stained with Sypro Ruby (Pierce). Three independent clones for each variant were measured. Variant names are indicated above the gel lanes. Also shown are molecular weight standards (M); negative control samples (C); BSA mass standards (Stds). Red arrows indicate positions of full-length phi29 DNA polymerase (top panel) or scFv (bottom panel). BSA standard lanes include 500, 250, 125, 62.5, and 25 ng total protein (top panel, left to right) or 1000, 500, 250, 125, and 50 ng total protein (bottom panel, left to right).

Gene variants were synthesized, sequence-verified and cloned under control of the T7 promoter (Materials & Methods). Expression of full-length protein was directly measured by polyacrylamide gel electrophoresis for a minimum of three clones for each gene variant (see Fig. 1). Sequences and expression levels for all genes in this study are provided in Table S1. Protein yields varied from undetectable (<5 µg/ml of culture at A_600_ = 3.0) to ∼68 µg/ml (estimated to be ∼10% of total cell protein) for the polymerase, and to ∼200 µg/ml (∼30% of total cell protein) for the scFv. Synonymous codon changes thus caused more than 40-fold differences in expression.

### Expression variation is caused by distributed sequence differences

Sequence characteristics affecting expression could be local (*e.g*. mRNA structures and rare codon clusters) or global (*e.g.* codon usage and GC content). To distinguish between localized or distributed effects, four sets of chimeras were constructed. For each set, a well-expressed gene and a poorly-expressed gene were divided into three segments (5′, middle and 3′), chimeras were synthesized and their expression determined (Table 1).

**Table 1:** Expression of 4 chimeric gene sets.

		A:	P15	P7	A17	A11
		B:	P19	P20	A1	A1
A	A	A	0.09±0.02	0.24±0.03	0.22±0.07	nd
B	A	A	0.13±0.02	0.19±0.03	0.32±0.05	0.66±0.14
A	B	A	0.09±0.01	0.48±0.02	0.31±0.05	nd
A	A	B	0.22±0.07	0.18±0.02	0.74±0.18	nd
A	B	B	0.43±0.03	0.56±0.03	0.85±0.15	
B	A	B	0.45±0.05	0.32±0.03	0.91±0.16	
B	B	A	0.32±0.04	0.79±0.05	0.28±0.02	
B	B	B	1±0.29	1±0.23	1±0.12	1±0.12

Expression levels are normalized to that of the highest expressing parent of each chimera set. Crossover points between three contiguous gene fragments for each set were as follows: For the P15/P19 chimera series, genes were split after codons 75 and 325 of the 575-codon polymerase genes. For P7/P20 chimeras, genes were split at codons 25 and 300. For A17/A1 and A11/A1chimeras, genes were split at codons 43 and 163 of the 281-codon scFv genes. “nd” indicates that expression was not detected. Standard errors for a minimum of three replicates are shown.

Chimeras between polymerase genes P19 (high expresser) and P15 (low expresser) showed highly distributed effects. The 5′ or 3′ segments of P19 when individually substituted into P15 increased expression over that seen for P15 alone, with the strongest effect contributed by the 3′ segment. Conversely, any one segment of P15 was deleterious when substituted into P19, with the strongest effect again seen for the 3′ segment. In contrast, expression of chimeras of P7 (low expresser) and P20 (high expresser) showed a strong dependence on the parental origin of the middle segment with relatively little contribution from the 5′ and 3′ segments. Chimeras between scFv genes A1(high expresser) and A17 (low expresser) correlated strongly with the parent of the 3′ segment, while chimeras between scFv genes A1 and A11 (low expresser) expressed protein at comparable levels to the parent of the 5′ segment (first 43 codons).

These results show that protein expression levels can depend on elements distributed throughout the gene and are not confined to any specific region. Each of A11, A17 and P7 appeared to have a dominant “poisoning” segment; replacement of that segment with the corresponding segment from a good expresser boosted expression close to the level seen in the good expresser. However the position of the deleterious segment was different in each of these chimera sets, while in the P15/P19 set each segment contributed similarly.

We analyzed all gene variants, attempting to correlate expression levels with properties that have been suggested to affect expression (see Table S1). We could not find correlation with previously suggested deleterious motifs, such as predicted 5′ or internal mRNA secondary structures, GC bias in the first 15 codons, content, runs of C or G, transcriptional terminator motifs [20], [21], internal Shine-Dalgarno-like motifs [22], RNaseE cleavage sites [23], or over- or under represented codon pairs [24], [25]. This suggested that expression level differences were either determined by several unidentifiable elements or were influenced by a distributed sequence property such as codon usage.

### Synonymous codon choice correlates with expression

**Figure 2:**
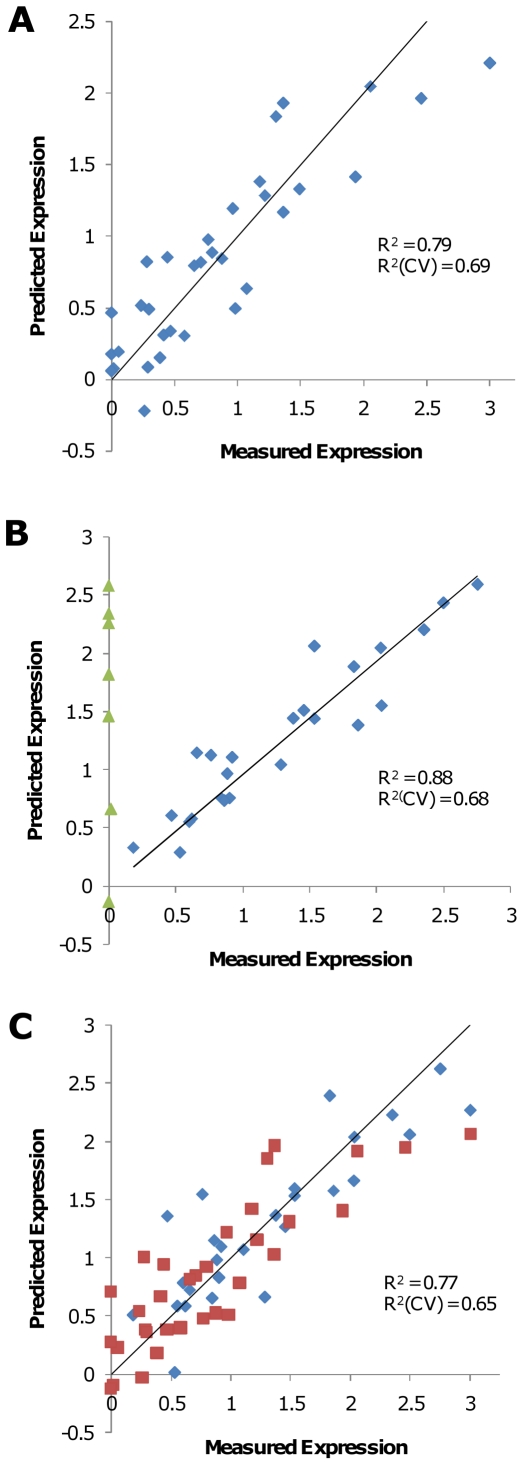
PLS codon frequency models. For each variant the measured expression level was plotted against the expression predicted from a PLS model using genetic algorithm-selected codons. (A) Model fit for polymerase variant expression data. Blue diamonds indicate the 34 gene training set used to create the model. (B) Model fit for scFv expression data. Blue diamonds indicate the 24 gene training set used to create the model. Green triangles are variants from the initial set with undetectable expression, and which were not used for model building. (C) Combined model constructed from polymerase variants (34 red squares) and scFv variants (27 blue diamonds). Expression in each set was normalized to the highest expression level in that set ( = 3). R^2^(CV) indicates the correlation coefficient for the fit of the model in cross-validation (see Materials and Methods). Variants used to provide datapoints for construction of the models are indicated in Table S1.

Models to predict expression as a function of codon usage were constructed using Partial Least Squares (PLS) regression [15]. Models were calculated from the polymerase and scFv variant sets separately and in combination. Initial regression analysis with all sense codons suggested that frequencies of only a subset of codons could explain most expression variation. A genetic algorithm was used to evolve 888 highly-predictive unique PLS models, each with a reduced set of codons (average of 14.2 codons per model). The predictions of the best models are shown in Figure 2.

Using on average 80% of the 34 datapoints in the polymerase set, the model predicted expression from the remaining 20% of sequences with a correlation coefficient of 0.69 (Figure 2A). Genetic algorithm selection identified 6 codons representing biases for 6 different amino acids (Gly, Leu, Asp, Glu,Tyr, and Ala) as most critical for modeling. Five of these 6 codons (all but Ala) encode the 5 most utilized amino acids in the polymerase and 5 of 6 (all but Leu) encode amino acids that are utilized at levels above that of average genes in *E. coli*.

Initial modeling of scFv expression gave results similar to the polymerase. However, 9 scFv gene variants showing no or barely detectable expression were difficult to explain along with the remainder of the set. When these 9 variants were not included in the model, a strongly predictive model was obtained (R^2^ = 0.86, Cross-Val R^2^ = 0.68; Fig. 2B). Genetic algorithm selection identified 5 codons representing 4 amino acids (Ser, Thr, Ala, and Val) as most significant.

The two datasets were combined by normalizing expression of each gene to the highest expressing gene in its set. A PLS model of the combined data is shown in Figure 2C (R^2^ = 0.77, Cross-Val R^2^ = 0.65). Fitting statistics for this model are shown in Table S3. As a further validation of low sensitivity to over-fitting of our method and significance of the correlations observed, the genetic algorithm was applied to datasets where the association of expression levels to variants was randomized. No predictive model based on codon frequency could be obtained after randomization for either the polymerase dataset (Best model: R^2^ = 0.037, Cross-Val R^2^ = 0.004), the reduced scFv dataset (Best model: R^2^ = 0.44, Cross-Val R^2^ = 0.30) or the combined dataset (Best Cross-Val R^2^ = 0.11). We conclude that variation in expression within our dataset is highly correlated to codon usage.

### Preferred codons are not those used most frequently by *E coli*

Ten amino acids were consistently represented in all 888 evolved models (Table 2), suggesting they are critical for optimal prediction. All other amino acids were represented in fewer than 30% of the models. Summary codon usage data for the 10 highly-represented amino acids is shown in Table 2. The codon bias observed for highly expressed genes of the dataset is different from codons used at highest frequency in naturally highly expressed *E. coli* genes [5], [7]. We also see no correlation between codon bias of highly expressed native *E. coli* genes and the codon regression vectors obtained from PLS regression of our data (Table S3). For example, although Ser-UCU is preferred in highly expressed *E. coli* genes, our model indicates that Ser-AGC is preferred: it is used 7 times more often than UCU in our most highly expressing genes. For threonine, highly-expressed *E. coli* genes use ACC 4 times as often as ACG; our model suggests that ACG should be used more often than this, and our most highly expressed variants use ACG at over half the frequency of ACC.

**Table 2:** Codon biases that predict expression in PLS models of the combined datasets.

AA	Codon	GA Incl.	*F*_c_, HE_coli	*F*_c_, Dataset	*F*_c_, Best Variants	%A, scFv	%AA, Pol	%A, coli	tRNA sensitivity
Ala	GCA	1.00	0.24	0.17	0.24	6.4	4.5	10.2	1.9
Ala	GCC	0.07	0.16	0.12	0.19				21.5
Ala	GCG	0.38	0.32	0.47	0.44				1.9
Ala	GCU	0.90	0.28	0.24	0.12				2.0
Gly	GGA	1.00	0.02	0.03	0.00	11.7	7.1	8.2	0.6
Gly	GGC	0.00	0.43	0.41	0.39				12.5
Gly	GGG	0.07	0.04	0.03	0.00				0.3
Gly	GGU	0.02	0.51	0.53	0.60				12.5
Phe	UUC	0.43	0.71	0.55	0.55	3.2	5.2	3.3	NA
Phe	UUU	0.68	0.29	0.45	0.45				NA
Ser	AGC	1.00	0.24	0.32	0.68	12.5	4.2	4.7	3.4
Ser	AGU	0.06	0.04	0.05	0.00				3.4
Ser	UCA	0.01	0.05	0.05	0.02				7.5
Ser	UCC	0.20	0.27	0.23	0.13				35.5
Ser	UCG	0.00	0.07	0.08	0.05				4.4
Ser	UCU	0.05	0.33	0.27	0.10				7.9
Lys	AAA	0.27	0.79	0.68	0.51	3.9	11.3	6.5	NA
Lys	AAG	0.93	0.21	0.32	0.49				NA
Pro	CCA	0.19	0.15	0.13	0.10	3.6	3.8	3.9	32.0
Pro	CCC	0.02	0.02	0.03	0.00				2.1
Pro	CCG	0.86	0.72	0.75	0.81				13.3
Pro	CCU	0.04	0.11	0.08	0.09				3.7
Asp	GAC	0.43	0.54	0.50	0.54	6.0	7.3	5.8	NA
Asp	GAU	0.74	0.46	0.50	0.46				NA
Leu	CUA	0.00	0.01	0.01	0.00	7.5	7.7	8.5	26.9
Leu	CUC	0.00	0.08	0.05	0.03				24.8
Leu	CUG	0.16	0.77	0.68	0.78				5.5
Leu	CUU	0.70	0.06	0.05	0.02				24.8
Leu	UUA	0.06	0.03	0.06	0.03				3.0
Leu	UUG	0.54	0.05	0.15	0.14				0.6
Gln	CAA	0.69	0.19	0.31	0.45	5.0	2.3	3.5	32.2
Gln	CAG	0.35	0.81	0.69	0.55				14.4
Thr	ACA	0.14	0.04	0.05	0.00	8.2	6.3	5.4	5.7
Thr	ACC	0.00	0.54	0.61	0.57				20.9
Thr	ACG	0.99	0.13	0.14	0.33				2.4
Thr	ACU	0.00	0.29	0.19	0.10				6.6

A genetic algorithm was used to identify codon biases that best explained expression levels for the combined datasets. The algorithm evolved 888 unique codon subsets with root mean square error in cross-validation within 5% of that of the best predictive subset. These evolved subsets contained an average of 14 codons each. The codon biases for 10 amino acids were represented by at least one codon in greater than 99% the majority of the subsets. All other amino acids were represented at less than 30% in the subsets. Codon frequency data for the 10 highly represented amino acids is shown. Column 3 (“GA incl.”), frequency of inclusion of the specified codon in the 888 selected subsets. *F*_c_, codon usage frequency per cognate amino acid, shown for a subset of naturally highly expressed *E. coli* genes (“HE_coli”) [5], for the entire combined dataset (“Dataset”), and for 10 highly expressed genes among the dataset (“Best variants”; see text). %AA, percent usage of the indicated amino acid in the scFv and polymerase (“Pol”) genes as well as that measured for the E. coli proteome (see Materials and Methods). “tRNA senstitivity” is an estimate of the sensitivity of the charged cognate tRNA supply to amino acid limitation [36].

**Figure 3:**
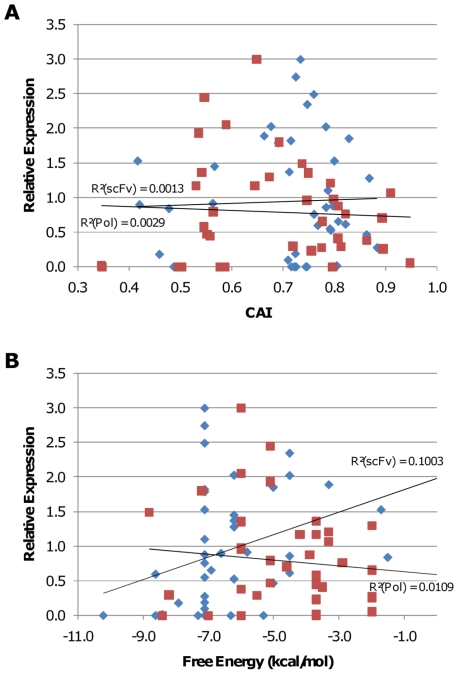
Expression is not predicted by Codon Adaptation Index or mRNA structure. The codon adaptation index [7] (part A) and the strength of mRNA secondary structure from position −4 to +38 relative to the initiating AUG (part B) were calculated for each variant synthesized in this study and plotted against the expression level measured for that variant. Blue diamonds indicate scFv variants. Red squares indicate polymerase variants. Expression levels are normalized to highest expressing variant for each set (equal to 3).

Bias towards codons that are most used in highly expressed native *E. coli* genes (increasing the CAI [7]) is widely used as the basis of gene optimization [26]. The discrepancy between these codons and those that our PLS model indicates are important is therefore significant. Plotting the CAI score against the expression obtained for each gene in our study confirmed that CAI has no value in predicting gene expression for either gene set (Fig. 3A).

Another factor that has recently been shown to be important for expression of green fluorescent protein is the structure of the mRNA around the initiating AUG [11]. For the most part, we see no correlation between the energy of RNA structure in this region and expression (Fig. 3B), although a few of the most structured scFv genes show very poor expression that was not predicted by our codon usage model.

### 5′ mRNA affects expression in some scFv variants

**Figure 4:**
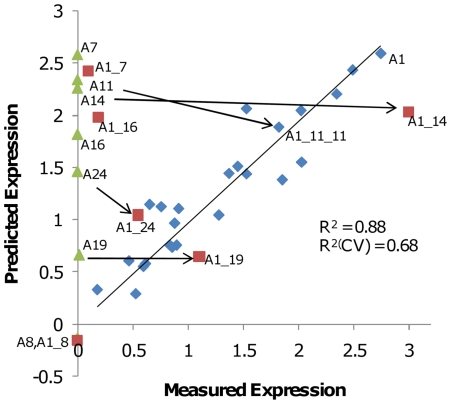
Modification of 5′ sequence improves the performance of some scFv variants. For each scFv variant the measured expression level was plotted against the expression predicted from a PLS model using genetic algorithm-selected codons. Blue diamonds indicate the 24 gene training set used to create the model, Green triangles are variants from the initial set with undetectable expression. Red squares are new variants created by combining the first segment (the first 15 codons) of variant A1 with the remainder of these 6 poorly-expressed variants. Arrows indicate changes in predicted and measured expression upon 5′ codon exchange. Variants represented as green triangles or red squares were not included in the training set from which the model was built. Variant A1_11_11, in which a larger 43 codon portion of the 5′ section of the A11 gene was replaced with that of A1, is also indicated for comparison.

From the analysis expression levels of the variant chimeras, we observed that the 5′ segment of A11 appeared highly deleterious for expression (Table 1). This poorly expressing variant is predicted to express highly based on our codon usage model. Several reports have implicated the 5′ coding region as especially important in modulating translation initiation [3], [11], [27]–[33]. A recent study implicated mRNA structure formed in the region from −4 to +38 relative to the start of the ORF (starting A identified as position 1) as particularly deleterious for green fluorescent protein expression [11]. The 5′ regions of variants A11, A14, A16, and A19, all of which expressed at levels below those predicted by our codon usage model also showed stronger than average predicted mRNA secondary structure around the site of translational initiation (Table S1). We therefore tested whether replacing the 5′ segment of 6 other antibody genes would increase protein expression levels as it had for A11. Three of 6 of the non-expressing antibody genes were clearly improved when their 5′ segments (the first 15 codons) were replaced by that of A1. An A1_14 chimera expressed at similar levels to A1. Chimeras A1_19 and A1_24 showed improved but lower expression than A1_14. Barely detectable expression was seen for the A1_7 and A1_16 chimeras and no expression was seen for chimera A1_8, indicating that something other than the 5′ leader is limiting expression of these gene variants. Four of these 6 gene variants (all that show significantly improved expression and A1_8) are well predicted from their codon usage by the PLS model, as shown in Figure 4.

None of three weakly expressing polymerase variants (P2, P11, and P16) were detectably improved by exchange of the first 15 codons with those of the highly expressed P19 (data not shown). Clearly, these genes are poor due to downstream elements or global features. All three were predicted to be low expressed based on codon usage by the PLS model.

### Variation among gene chimeras is largely explained by codon usage

**Figure 5:**
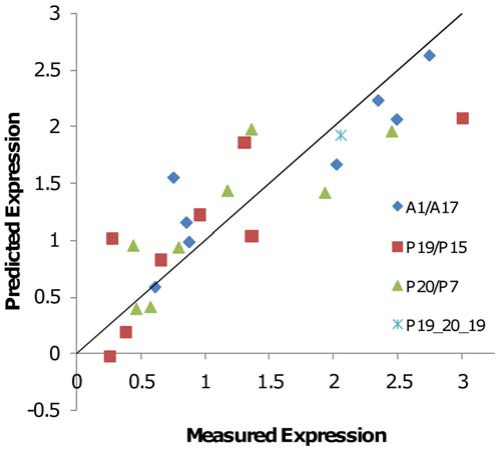
Prediction of variant chimera expression by the combined dataset PLS model. Expression predicted by the combined model shown in Figure 2C for the subset of chimeric variants. Each chimera series is indicated by different symbols as shown in the legend.

The variation in the distribution of critical sequence determinants among variant gene chimeras is described in Table 1. Chimeras between polymerase variants P15 and P19 showed that expression levels resulted from sequence properties that were distributed throughout the gene, whereas other chimera sets showed different emphasis on particular gene fragments. With the exception of the chimeras made with one parent showing undetectable expression, indicating a deleterious 5′ mRNA leader, the variation among the chimeras is largely explained by the codon usage based PLS model, as shown in Figure 5.

We interpret these results to mean that codon usage is a strong determinant of the overall expression level that can be obtained from a gene, but that this level can be reduced if deleterious sequence elements are present, for example those that form mRNA structures that may interfere with the initiation of translation.

### Codon usage models supported by design and testing of new gene variants

Although the PLS model uses codon usage to predict gene expression for two different gene sets, it does not directly provide an optimal codon usage. Rather it indicates which codons should be used more and less often than their average use in the dataset (see Table 2). An optimal solution from this kind of experiment typically requires several iterations of modeling and testing [34], but this is unnecessary to demonstrate that expression depends upon the frequencies of particular codons. Instead, we tested this hypothesis by designing a set of new genes using new codon bias tables, measuring their expression and comparing with that predicted by the model.

**Figure 6:**
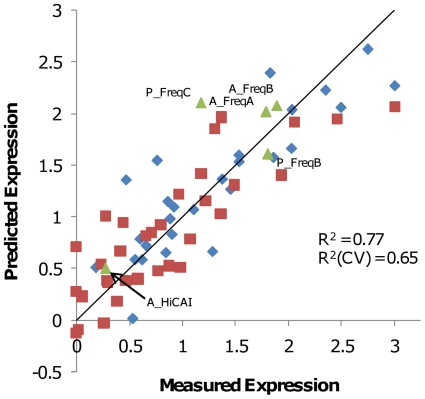
New gene variants express as predicted by the combined PLS model. For each variant the measured expression level was plotted against the expression predicted from a PLS model using genetic algorithm-selected codons. Polymerase variants (34 red squares) and scFv variants (27 blue diamonds) were included in the training set, expression in each set was normalized to the highest expression level in that set ( = 3). Green triangles show measured and predicted expression of 5 new genes not included in the training set. Correlation coefficients represent fits of the entire training set.

We tested three different codon biases, none of which we expected to be optimal, but all predicted to give better than average expression in the dataset. These frequencies are shown in Table S2. The expression of 2 scFv variants (A_FreqA and A_FreqB) and 2 polymerase variants (P_FreqB and P_FreqC) synthesized using these tables are shown in Figure 6, and their expression levels are also given in Table S1. All of these genes expressed extremely well, and as predicted by the PLS model. In contrast, an additional scFv variant, A_HiCAI, was designed using codons that occur frequently in highly expressed native *E. coli* genes. This gene also used identical coding to the highest expressed variant A1 for the first 15 codons to avoid possible deleterious mRNA structure near the translational initiation site. Known toxic motifs were avoided in the design. A_HiCAI's expression, accurately predicted by the PLS model, was only 15% of the levels obtained for A_FreqA or A_FreqB. This data supports the conclusion that controlling gene codon frequencies, but not maximizing CAI, is critical for optimal protein expression.

## Discussion

By designing and synthesizing 81 individual genes encoding two different proteins, we have found that sequence differences entirely confined to non-coding changes within the open reading frame caused at least 40-fold differences in protein expression. We were able to create predictive sequence-expression models based on a strong correlation between expression and the codon bias of a subset of amino acids. The model correctly predicted the expression of variants not included in the model-building, and of new variants designed using improved codon bias tables.

Most of the codons that were identified as influential for expression encode amino acids that are highly represented in one or both proteins studied (Table 2). However, the most favorable biases for expression clearly do not correspond to those found in highly expressed native *E coli* genes [5], [7]. This contradicts a widespread gene design principle that mimicking the codon bias of the host or of a selected group of host genes will ensure protein expression [6], [26]. The rationale for this approach has been that tRNA availability could limit translational elongation. However, translation is not limited directly by tRNA levels, but by the availability of amino-acylated (charged) tRNA [35].

In 2003, Elf *et al* [36] predicted that charging of some tRNA isoacceptors would be much more sensitive than others to perturbations of the recharging rate. These are tRNAs used at high frequency relative to their level in the cell. Subsequently, these predictions were experimentally confirmed for a subset of tRNAs [37]. Furthermore, heterologous overexpression is predicted to deplete intracellular amino acid and charged tRNA concentrations depending on the amino acid composition of the overexpressed protein [38], [39]. This may have a direct impact on translation rate and may also induce metabolic responses deleterious for expression yield [38].

PLS modeling suggests that most of the variation in our dataset can be explained by codons for serine (AGC favored and UCU disfavored), threonine (ACG favored), and leucine (UUG favored). These results fit well with the predicted sensitivities to amino acid starvation of the isoacceptor tRNAs that recognize these codons [36]. The tRNA pools for all three favored codons (AGC, ACG and UUG) are the least sensitive to starvation for their respective amino acids (Table 2). The relative tRNA charging levels during starvation have been measured for threonine and leucine [37]. From this data and from the tRNA abundance [40] we can estimate the number of copies of each charged and uncharged tRNA per cell (Table 3). Considering either absolute numbers of charged tRNAs or the ratio of charged to uncharged tRNAs, UUG becomes a more attractive codon for encoding leucine relative to CUG as recharging is limited by starvation. Likewise ACG improves greatly relative to ACC for encoding threonine. Both trends are consistent with the codon preferences identified by our PLS model.

**Table 3:** Charging of leucine and threonine codons under starvation conditions.

Codon	tRNA^1^	# per cell^1^	# per codon^1^	Relative charging^2^	# Charged	Total # Charge per codon	Total % charged
CUG	Leu1	4470	5136	0.086	384	410	8
	Leu3	666		0.039	26		
UUG	Leu4	1913^3^	2944	0.24	459	676^3^	23
	Leu5	1031^3^		0.21	217		
ACG	Thr2	541	1457	0.2	108	190	13
	Thr4	916		0.09	82		
ACC	Thr1/3	1020	1020	0.081	83	83	8
ACA	Thr4	916	916	0.09	82	82	9
ACU	Thr1/3	1020	1936	0.081	83	165	9
	Thr4	916		0.09	82		

Charging of tRNAs and the codons that they recognize calculated from published data.

1 From Dong, et al [40].

2 From Dittmar, et al [37].

3 We note that discrepancy between studies in estimates of total tRNA for Leu4 and Leu5 have been reported [48].

From this data it is tempting to speculate that much of the variation we see in expression is influenced by charged tRNA depletion and/or induction of a metabolic response from the host organism. High translation rates deplete the translational machinery [41]. As amino acid charging of tRNA becomes limiting, only those tRNAs that can maintain charge can support high translation levels. The optimal codon bias for a gene probably depends both on maintaining high levels of charged tRNAs and minimizing the levels of uncharged tRNAs which may inhibit translation and/or cause a deleterious metabolic response [38], [39], [41].

In contrast with a recent study of GFP variants [11], we saw relatively little influence of mRNA structure near the initiation site. In three scFv genes weak expression, poorly predicted by the model, correlated with stronger than average mRNA structure in this region. Replacing the first 15 codons with a less-structured synonymous equivalent restored expression to levels predicted by the model, suggesting that mRNA structure may limit expression of these genes. In reconciling our results with those of Kudla et al, we note that the predicted 5′ mRNA structures of almost all of our genes are significantly weaker than those found to have a significant effect in the GFP study: only one of our gene variants had a free energy less than −9 kcal/mol in this region (Table S1). Indeed, little correlation was observed in the GFP study between 5′ mRNA structure and expression for genes with structure strength >−9 kcal/mol despite greater than 20-fold variation in expression among these genes [11]. Inhibition of initiation by especially strong mRNA structure would obscure effects resulting from factors that influence elongation, such as codon usage, which dominates our results.

Although we were unable to find any predictive correlations between expression and any parameter other than codon frequency, other sequence elements may contribute to some variation observed and could be important in optimal gene design. Differences in mRNA stability could also cause at least some expression variation observed. The translation rate itself can influence mRNA degradation rate making cause and effect in this case difficult to disentangle [42]–[44].

As direct synthesis replaces classic cloning as the preferred path for constructing functional genetic elements, it is critical to develop gene design algorithms for reliable heterologous expression. Here we have shown that sequences beyond the translational initiation region are critical and that codon usage is a key determinant of expression yield. Regardless of the mechanism by which codon bias affects expression, systematic analysis of the relationship between gene sequences and expression will be a powerful tool to refine our design algorithms, both for *E. coli* and other expression hosts.

## Materials and Methods

### Gene synthesis and protein expression

Synthetic gene variants and chimeras were all made by standard in-house procedures essentially as previously described [45] and cloned into a pET24a expression vector (EMD, Madison, WI) under control of the T7 promoter, between the XbaI and EcoRI restriction sites. Each construct was completely sequenced in both directions to ensure consistency with the designed sequence. Each variant plasmid was transformed into *E. coli* expression host strain BL21(DE3) pLysS (Invitrogen, Carlsbad, CA). BL21(DE3) pLysS was chosen as the host for all expression studies described. The low-level expression in this host of T7 lysozyme, an inhibitor of T7 RNA polymerase, gives tight repression of heterologous expression prior to induction to minimize potential gene toxicity which could affect data quality.

Prior to protein expression analysis of the variants, expression was analyzed for multiple variants showing a range of expression levels to determine appropriate expression time and temperature. Strong, consistent expression was achieved at 30°C, a commonly used temperature for heterologous expression in *E. coli*. Time courses at 30°C showed expressed protein levels increasing to a maximum after approximately two hours, as the cells entered stationary phase growth, and expression remained steady for at least five hours. Relative protein expression levels between these variants were consistent as protein accumulated during growth phase and were maintained in stationary phase (data not shown). For our variant analysis we chose to express for four hours at 30°C.

At least three independent isolates for each gene were picked and cultured overnight in 2 ml Luria Broth (LB) containing 25 µg/ml kanamycin and 25 µg/ml chloramphenicol. Overnight cultures were diluted 50-fold in fresh media and incubated at 37°C until the cells were in mid-log growth (OD at 600 nm = 0.6). Expression was induced by addition of IPTG to 1 mM and incubation for four hours at 30°C. Final optical densities of cultures were measured and equivalent amounts of culture were analyzed by polyacrylamide gel electrophoresis. Gels were stained with Sypro Ruby (Pierce), visualized by fluorescence imaging, and protein band intensities quantified using TotalLab100 image analysis software (Nonlinear, Inc). Each gel contained protein concentration standards to calibrate band intensity. In each experiment, a consistent reference variant was co-expressed in triplicate. For analysis of polymerase expression, the reference was a phi29 DNA polymerase variant identical to variant 21 but containing two differences in the 5′ untranslated region. For analysis of the scFv variants, Variant A13 was used as the reference. Reference variants were used to correct for experiment to experiment variation in yield. Measured expression levels are all relative to these references. Reported values in µg/ml are normalized to the average expression level of the references over the sum of experiments. The detection limit of the assay was approximately 5 µg protein per ml culture at an A_600_ = 3. The standard error of measured expression for variant repeats was generally <20% of the mean.

### Design of initial gene variant sets

Gene variants were designed by back-translating the protein sequence using a Monte Carlo repeated random sampling algorithm [19]. This algorithm selects a codon for each position at a probability defined in a codon frequency lookup table. A variety of different lookup tables and constraints were applied to create variant designs that differed in a number of parameters that have been associated with expression effects in the literature. Global codon usage was primarily varied in bias toward or away from codons used preferentially in either highly expressed or average native *E. coli* genes and inclusion or exclusion of naturally rare codons. We also specifically varied the first 15 codons toward higher or lower GC content. Design of Experiments methodology was used to minimize co-variation of these constraints and thus maximize diversity among the variants and improve our ability to distinguish independent contributions. We also analyzed and edited mRNA structure to minimize co-variation of structure, internally or near the translational initiation site, with codon usage constraints. We also minimized co-variation of codon bias with G/C islands, which might promote frame-shifting, by selectively avoiding runs of consecutive G and/or C greater than 6 nucleotides for half of the variants. Due to the use of Monte-Carlo sampling in the gene design, all of these variants were highly divergent in sequence identity from each other. The average pairwise DNA sequence identity was 79% within the 24 member scFv dataset and 82% within the 21 member polymerase dataset. This broadly distributed dataset ensures that most global sequence variables are sampled, either as a direct consequence of the design, or as an indirect consequence of the varied dataset. Except where restriction sites were fixed in the scFv genes, no contiguous string of nucleotides longer than five nucleotides was conserved throughout either set. With the exception of codons used extremely infrequently in *E. coli*, the frequency use of all individual sense codons showed high variability across the sets. The complete sequence alignment of both gene variant datasets is available in Table S1.

### PLS analysis and genetic algorithm variable selection

The frequency of occurrence of each codon was calculated for each gene variant and compiled for all variants that gave detectable protein expression. Stop codons were not varied in frequency and thus were not included in the modeling. For individual phi29 polymerase and scFv gene models, sense codons with no or redundant information content were excluded from the dataset. These included ATG for Met, and TGG for Trp and one codon for each two-codon amino acid, as, for such amino acids, the frequencies of the two codons are perfectly inversely correlated. For modeling of the combined dataset, all sense codons were included.

We used the PLS Toolbox 5.2 software (Eigenvector Research Inc., Wenatchee, WA) run in the MATLAB environment (Mathworks, Inc.) to model the relationship between sequence variables and protein expression data. For all modeling, independent (e.g., codon frequency) and dependent (expression) variables were pre-processed by mean centering and scaling to the standard deviation of variation among the samples included. For cross-validation, the dataset was randomly split into 5 subsets of variants and each subset was predicted by PLS models trained on the remaining 4 subsets. This process was repeated for ten iterations with different random data splits. The error in prediction of left-out data was monitored as a function of the number of latent variables used. In each case described, the number of latent variables used was limited to the number that minimized error in cross-validation models to avoid over-fitting. As an additional check of sensitivity to over-fitting, we randomly re-assigned expression data to the samples, such that original and randomized data were not correlated, and assessed the ability of PLS to construct a model. For all modeling described, such randomization prevented construction of any predictive model.

For variable selection a genetic algorithm was used to determine codon subsets that were most predictive of expression (those that minimized cross-validation error). For typical codon based modeling, 256 random codon subsets, each consisting of approximately 30% of the total codon pool, were chosen. PLS models were created for random cross-validation subsets of the samples as described above. Codon sets were then ranked in fitness according to their cross-validation error in prediction of expression. Variable sets that showed better than median error in prediction were chosen as parents and randomly pair-wise recombined to create new progeny subsets. Along with their parents, these were then evaluated for prediction and best subsets again selected. At each generation a low level of mutation (random substitution of variables at a frequency of 0.01) was allowed to avoid trapping in local optima. This process was continued until convergence, defined here as fewer than half of the subsets in the population being unique (i.e., ≤128 unique subsets in a population of 256). The entire evolutionary process was repeated 20 times to further avoid bias from local optima trapping, creating a population of codon subsets improved in prediction of expression. The same selection process was also run after random assignment of expression data as described above. In each case, randomization prevented the algorithm from identifying a predictive model. Results were used in to identify codons most enriched in the evolution, and thus most critical for explaining expression. The best evolved subsets were used to create working PLS models.

### Gene Sequence Analyses

All RNA structure strengths are optimal predicted structures calculated using the Vienna RNAfold software package using parameters from Mathews, et al [46]. CAI estimates were calculated as the geometric mean for test gene codons of the ratio of the codon frequency in highly expressed *E. coli* genes divided by that of the highest frequency codon for each amino acid in those genes [7]. Codon frequencies in highly expressed *E. coli* genes were taken from EMBOSS [47].

## Supporting Information

Table S1 Expression data, sequences, and selected sequence characteristics of all polymerase and scFv variants. Variants beginning with “A” are scFv genes. Those beginning with “P” are polymerase genes. Column 2 shows the calculated average expression level relative to the highest expressing variant for the same gene set (set to 3). Columns 3 & 4 show the calculated absolute expression level and standard error (minimum of 3 independent determinations). Columns 5–7 indicate which variants were included in datasets used for PLS modeling for the figures indicated. Columns 8 & 9 show the calculated GC content and codon adaptation index (CAI) [7], respectively, for each variant. Columns 10 & 11 show the number of occurrences of codons used at <10% per amino acid in naturally highly-expressed and all E. coli genes [5], [47], respectively. Column 12 shows the number of occurrences of contiguous runs of G and/or C of 7 or more nucleotides. Columns 13 & 14 show the strongest and average RNA structure strength for all 50 nucleotide frames along the mRNA open reading frame region. Columns 15 & 16 show calculated mRNA structure strengths for two 5′ mRNA windows, one including sequence from 5′ to the ribosome binding site to 50 nucleotides into the open reading frame (−42 to +50) and one identical to that shown to correlate best with expression of GFP variants [11]. All RNA structure strengths are optimal predicted structures calculated using the Vienna RNAfold software package using parameters from Mathews, et al [46]. Column 17 includes the complete DNA sequences of the variant open reading frames and those for the 5′ and 3′ untranslated regions listed in the bottom three columns of the table.
(0.14 MB XLS)

Table S2 Codon Frequency Bias Tables. Two codon frequency tables were constructed as averages of the frequencies found in the best variants: FreqA from the 4 genes comprised of the 2 most highly expressing variants of each set (A1, A21, P19, and P20); FreqB from 10 of the most highly expressing variants (P19, P20, A1, A21, A1_14, A17_17_1, A17_1_1, A1_17_1, A1_11_11, and A_FreqA) and FreqC approximates the bias used to create polymerase variant P19. A fourth set of frequencies, “HiCAI” used codons that are most common in highly expressed native E. coli genes.
(0.03 MB XLS)

Table S3 PLS model statistics for combined gene sets Statistics for the PLS model depicted in Figures 2C, 5, & 6 of the manuscript. Codons listed are those selected by genetic algorithm to provide minimal error in cross-validation for prediction of the dataset (see Materials and Methods). The particular model includes 5 latent variables, which were determined to yield minimal error in cross-validation. Mathematical descriptions of model statistics have been published by Eriksson, et al [15]. Statistics shown apply to the datatset preprocessed as described in Materials and Methods.
(0.03 MB XLS)
